# Blue and red in the protein world: Photoactive yellow protein and phytochromes as revealed by time-resolved crystallography

**DOI:** 10.1063/4.0000233

**Published:** 2024-01-31

**Authors:** Marius Schmidt, Emina A. Stojković

**Affiliations:** 1 Physics Department, University of Wisconsin-Milwaukee, 3135 N. Maryland Ave., Milwaukee, Wisconsin 53211, USA; 2 Department of Biology, Northeastern Illinois University, 5500 N. St. Louis Ave., Chicago, Illinois 60625, USA

## Abstract

Time-resolved crystallography (TRX) is a method designed to investigate functional motions of biological macromolecules on all time scales. Originally a synchrotron-based method, TRX is enabled by the development of TR Laue crystallography (TRLX). TR serial crystallography (TR-SX) is an extension of TRLX. As the foundations of TRLX were evolving from the late 1980s to the turn of the millennium, TR-SX has been inspired by the development of Free Electron Lasers for hard X-rays. Extremely intense, ultrashort x-ray pulses could probe micro and nanocrystals, but at the same time, they inflicted radiation damage that necessitated the replacement by a new crystal. Consequently, a large number of microcrystals are exposed to X-rays one by one in a serial fashion. With TR-SX methods, one of the largest obstacles of previous approaches, namely, the unsurmountable challenges associated with the investigation of non-cyclic (irreversible) reactions, can be overcome. This article describes successes and transformative contributions to the TRX field by Keith Moffat and his collaborators, highlighting two major projects on protein photoreceptors initiated in the Moffat lab at the turn of the millennium.

## DEVELOPMENT OF TIME-RESOLVED CRYSTALLOGRAPHY (TRX)

**FIG. 1.**
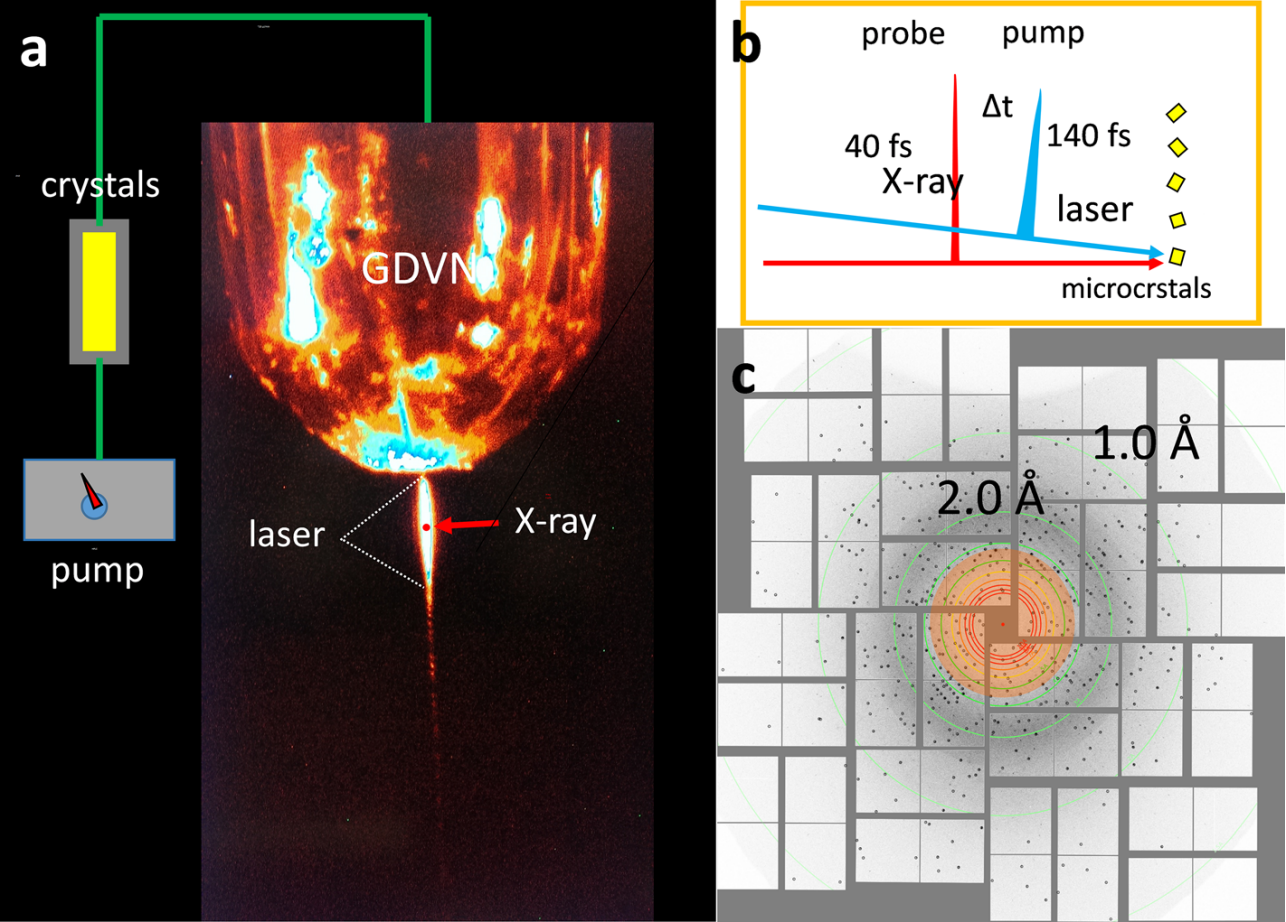
Time-resolved serial crystallography (TRSX) with the pump-probe method at free electron laser for hard x-rays (XFEL) light sources. (a) Basic setup. Microcrystals are flown into an injector device called “Gas Dynamic Virtual Nozzle”^28^ that generates a thin (∼5 *μ*m) jet. The reaction is initiated (pumped) by an intense, short laser light pulse (∼150 *μ*m in diameter) and probed by an x-ray pulse. (b) Schematic of the pulse sequence for a TR-serial femtosecond crystallography (SFX) experiment. The durations of the laser and x-ray pulses are marked. The reaction evolves by a time delay Δt before it is probed. (c) A diffraction pattern collected on photoactive yellow protein (PYP) microcrystals at the CXI instrument^29^ at the Linac Coherent Light Source (LCLS) with the Cornell-Stanford Pixel Area Detector (CSPAD).

The evolution of synchrotron light sources from the early use of particle storage rings^1^ to powerful 3^rd^ and 4^th^ generation synchrotrons was driving new opportunities with TRX. The biomedical importance of TRX is undisputed. For more than a century, researchers are highly interested in protein function focused on enzymes, which are catalytically active. Enzymatic activity plays a critical role in almost all diseases, pathogenic and/or hereditary in nature, including various infectious diseases and cancer.^2^ A large body of research exists to investigate biomolecular catalysis with time-resolved spectroscopic methods (TRSP). The pump-probe (PP) approach has been incredibly successful since it can be easily applied. Figure 1(b) shows the application of the PP approach with x-ray probe pulses. PP-TRSP merely requires a light source, a flashlamp (or a pulsed laser), and a data logger such as an oscilloscope or a computer card that charts the time-dependent output signal of a light sensitive detector which can be as simple as a photodiode. The Nobel laureates in Chemistry (1967), Porter, Norrish, and Eigen were pioneering this setup to time-resolve chemical reactions. Later, Gibson extended the same technique to biomolecules such as hemoglobin.^3,4^ TRSP reached spectacular sophistication with the appearance of the femtosecond laser (Nobel prizes for Ashkin, Strickland, and Mourou) and even attosecond laser pulses (Nobel prizes for Agostini, Krausz, and L'Huillier). Nevertheless, the relationship between the spectral response and the resulting structural change is rather unknown. This makes TR structure determination so important, as these methods provide a direct relationship between kinetics and the underlying structural changes. Moffat has been dedicated to achieving this in times where biomolecular structure determination was in its infancy.^5^ This way TR macromolecular crystallography is deeply rooted in the work of the early pioneers with the additional complication that much larger molecules with thousands of atoms are to be investigated.

**FIG. 2.**
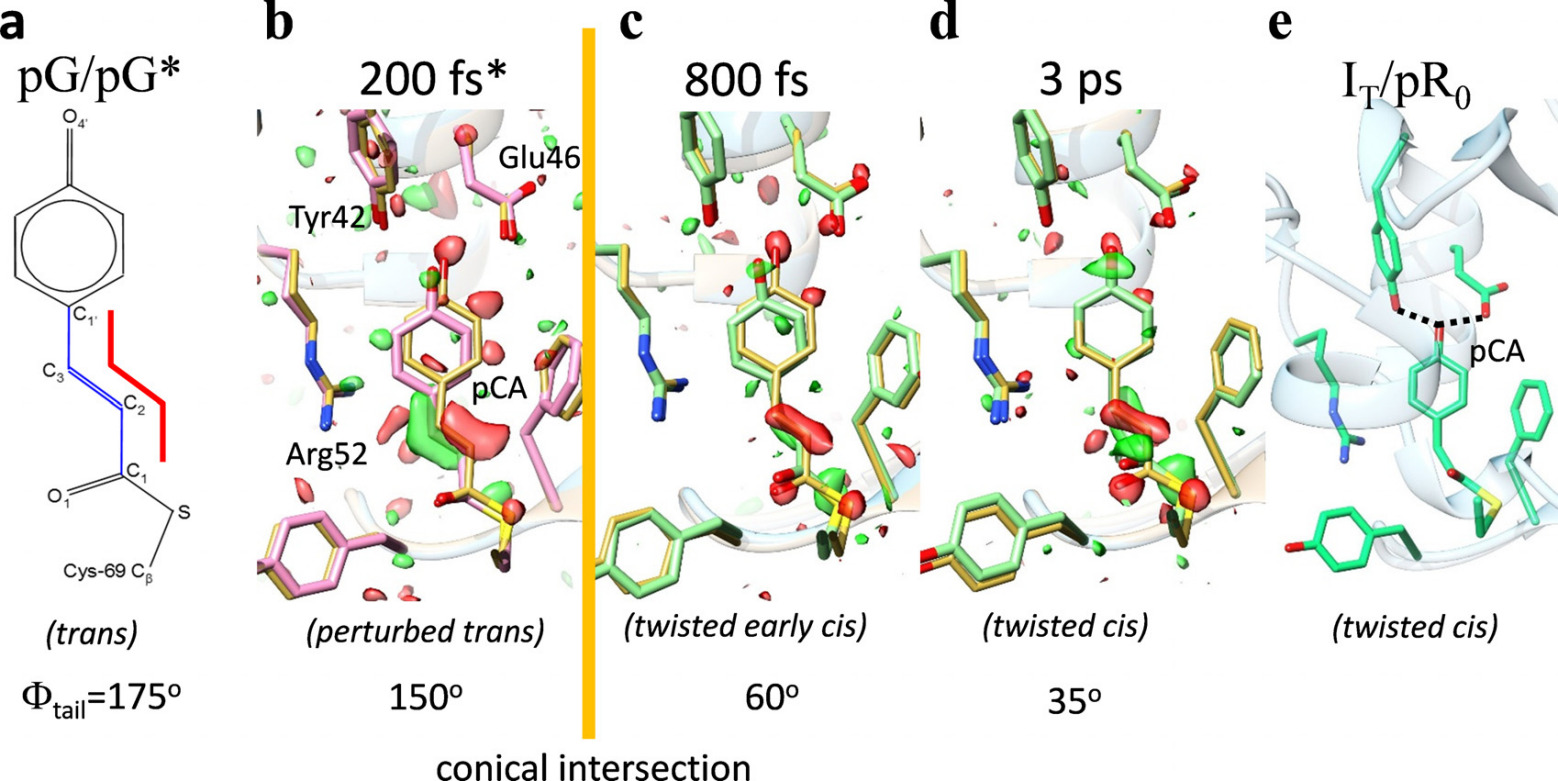
Femtosecond and picosecond dynamics of PYP as revealed by TRX. (a) The chemical structure of the pCA chromophore in the dark/reference structure (pG) and in the exited state (pG^*^) immediately after photon absorption. (b) Structure of PYP 200 fs after reaction initiation (pink). The star^*^ indicates relaxation on the excited state potential energy surface (PES). Yellow: structure of the reference/dark state. Some neighboring amino acids are marked. Negative and positive difference electron density features are shown in red and green (−3 sigma/3 sigma contour levels), respectively. Φ_tail_ is close to trans (150°). In (c) and (d), the situation 800 fs and 3 ps after light absorption is shown. The red and green difference density features change. Φ_tail_ is 60° at 800 fs and about 35° at 3 ps. The first stable intermediate (I_T_) is shown in (e) at around 150 ps.^47^

Only the integrated reflection intensities (IRI) are proportional to the structure factor amplitudes squared. Traditionally, the IRI are collected by rotating the crystals through the Ewald sphere. However, a time-resolved experiment requires that the IRI are collected quickly, faster than a millisecond (ms). Rotation of the crystal becomes impractical. Fast TRX was made possible by the application of Laue method^6^ to macromolecular crystallography. Using a broader bandwidth of the x-ray radiation, the IRI is collected instantaneously with TR Laue crystallography (TRLX). Moffat and colleagues developed this method with conceptual design,^7–9^ instrumentation development,^9–14^ data processing software solutions,^9,15^ data analysis algorithms,^16–20^ and pioneering experiments.^9,16,21,22^ Fundamental for the method are difference electron density maps^23^ that pinpoint positions where electrons have moved away illustrated by negative features, and others where electrons have moved to depicted by positive features [Figs. 2(b)–2(d)]. Details for difference map calculation and data analysis are described elsewhere.^24–27^ To stay ahead of the ever-evolving synchrotron light sources with new ideas and innovations to push the time-resolution of TRX to the limit must have been a sometimes frustrating, sometimes rewarding experience.

The time-resolution at synchrotron light sources is limited roughly by the duration of the x-ray pulses which is on the order of 100 ps. This time-resolution is sufficient to observe multiple processes in many macromolecular reactions. Fundamental reactions such as bond breaking and isomerization reactions, however, occur on a femtosecond timescale,^30–32^ that, consequently, cannot be examined. Particularly limiting was the inability to generate an analyzable diffraction pattern with a single, 100 ps x-ray pulse. Consequently, multiple exposures were overlayed on a detector before a diffraction pattern could be read out. This practice has been substantially changed due to two recent innovations:^33^ (i) the primary (incident) x-ray beam is shielded as much as possible from ambient air (or helium) by completely enclosing it, e.g., by a long hollow tube, except at the crystal position. In the best scenario, a detector with a central hole is available and the x-ray beam is stopped on the back side of the detector. The beamline will become essentially free of background scatter, caused by the highly intense, incident x-ray pulse. (ii) Photon sensitive, charge-integrating detectors such as the Jungfrau detector are used to capture very low reflection intensities. (i) and (ii) together make it possible to probe microcrystals with ever shorter synchrotron x-ray pulses up to the single pulse. Small crystals are exposed in a serial fashion to X-rays by viscous matrix injection or by quickly scanning using solid supports such as silicon chips or simple sheet-on-sheet foil fixed-targets.^33,34^

## SERIAL CRYSTALLOGRAPHY

With XFELs coming online, tiny crystals can be investigated. Every crystal that is exposed to the intense XFEL pulses becomes critically damaged. However, damage is a slow process relative to the duration of the x-ray pulse. A diffraction pattern is collected before the crystal disintegrates. This is called the diffraction-before-destruction principle.^35–37^ Since the crystal is damaged, another crystal must be provided. A dataset consists then of the diffraction form tens of thousands of tiny crystals that are exposed to the X-rays one-by-one, in a serial fashion^38–40^ (Fig. 1). It is serial crystallography, and not so much the diffraction-before-destruction principle, that revolutionized TRX. Born at XFELs out of necessity to replenish damaged crystals, serial crystallography is now also applied at synchrotron light sources.^33,34,41–44^ Although at XFELs a crystal size limit does not exist, it is an experimental observation^45,46^ that the pulse intensities are very often not sufficient to reach highest resolution with protein crystals that are below a certain size limit, say 1 or 2 *μ*m. To achieve acceptable resolution, larger (thicker) crystals are required that do not necessitate tightly focused x-ray beams. With larger crystals the x-ray focus can be widened, more volume is exposed and the crystals might not be damaged beyond acceptable limits.

Here, TRX experiments on two light-triggered macromolecular systems are highlighted. These are the photoactive yellow protein (PYP), a bacterial, blue-light sensing protein and the phytochromes, which are complex, red-light triggered enzymes with homologs in plants, fungi, and bacteria. They root on fundamental experiments performed in the lab of Keith Moffat and his collaborators around the turn of the millennium.

## TRX EXPERIMENTS ON PYP

**FIG. 3.**
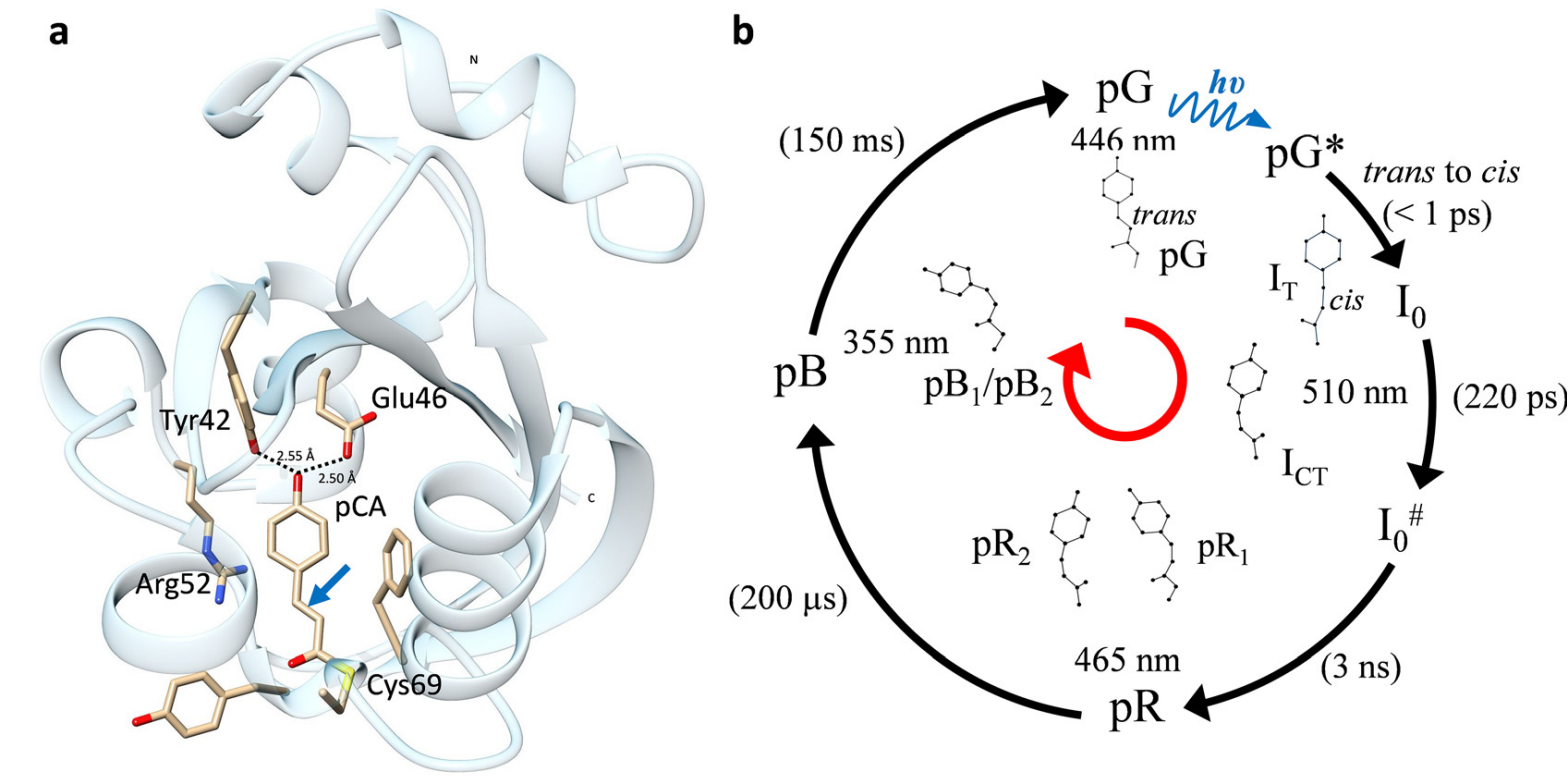
PYP structure and the corresponding photocycle. (a) The structure of PYP in its dark/reference state (pG). The central pCA chromophore and surrounding residues are displayed. The pCA is covalently attached to Cys69. In the dark, the pCA head is fixed by hydrogen bonds to Glu46 and Tyr42. The double bond about which the chromophore isomerizes is shown by a blue arrow. (b) PYP Photocycle. Nomenclature of structural intermediates from pG to pB is applied according to Jung *et al.*, 2013. Structures of the PCA chromophore are shown and related to spectroscopic intermediates (I_0_ to pB). Absorption maxima and approximate time scales are indicated. The *trans* to *cis* isomerization occurs on ultrafast time scales following the excitation of the chromophore. pG^*^ relaxes on the electronic excited state potential energy surface (PES) toward a conical intersection where isomerization occurs. The remaining photocycle proceeds on the electronic ground state PES.

PYP was discovered in 1985.^48^ Subsequently, its photoactivity was characterized,^49,50^ it has been overexpressed in *Escherichia coli*,^51,52^ and its 3D structure has been solved^53^ [Fig. 3(a)]. PYP is exquisitely well suited for TRLX since (i) it forms very well diffracting, stable crystals and (ii) it features a photocycle with numerous intermediates driven by a *trans* to *cis* isomerization of its central chromophore, para-coumaric acid (PCA) [Fig. 3(b)]. This way a unique opportunity exists to observe a photocycle together with a genuine, chemically highly important *trans* to *cis* isomerization in real time. In 1996, the first single pulse TRLX study was published by the Moffat group, at the time on the photolysis of carbon monoxide (CO) from CO-myogobin^22^ with a 4 ns time-resolution. PYP was the next logical candidate. The PYP photocycle [Fig. 3(b)] is initiated by the absorption of blue light and features several intermediate states. The structure of a longer lived intermediate, presumably PB_1_ or PB_2_, has been solved in 1997.^16^ In the following 15 years, the entire photocycle was characterized.^16,47,54–56^ This was made possible by an upgrade of the Laue beamline BioCARS^12^ at the Advanced Photon Source in Argonne National Laboratory that now allowed the (semi) automatic collection of entire time-series of Laue data from a single crystal. Of particular importance was the application of the singular value decomposition (SVD) to the x-ray data^18^ that allows the determination of the number of (distinguishable) processes in the reaction together with a compatible candidate chemical, kinetic mechanism, as well as the extraction of the difference electron densities of the intermediates. The structures of six intermediates called I_T_, I_CT_, PR_1_, PR_2_, PB_1_, and PB_2_ [Fig. 3(b)]^47,54,55^ were determined that provided insight how the *photocycle* proceeds. In short, the pCA chromophore in the earliest intermediate (I_T_) is already in a (twisted) cis configuration^47,56^ [Figs. 2(e) and 3(b)]. Two more intermediates (pR_1_ and I_CT_) are created from I_T_ by a hula-twist (HT) and bicycle pedal (BP) motion, respectively.^47^ I_CT_ reacts to pR_2_. pR_1_ and pR_2_ both react to the pB state. There is evidence that the PYP partly unfolds in solution.^57^ This view is corroborated by 5D crystallography (5DX),^58^ which is able to determine barriers of activation between some of the states. In solution the pB to pG transition is controlled by a large negative entropy (ΔS is about – 200 J/mol/K) of the transition state.^59^ This means that the transition state is much more ordered (refolded) compared to a disordered (more unfolded) pB state. In crystals, the barrier of activation determined from 5DX data is largely enthalpic and features a small, positive entropy (ΔS = 40 J/mol/K).^60^ This shows that the transition starts from a well ordered pB state and continues to the final pG state through a more flexible transition state where the chromophore is only loosely bound presumably in its binding pocket.

The *trans* to *cis* isomerization itself could not be time-resolved at synchrotron light sources. This changed when the first XFEL, the Linac Coherent Light Source (LCLS), came online. Although the XFEL can be viewed as the ultimate machine for TRX experiments, it was considered initially unsuitable for TRX experiments due to (i) limited spatial resolution reached after single x-ray pulse exposure and (ii) inferior data quality caused by the SASE jitter (where SASE stands for x-ray generation by self amplified spontaneous emission) of both the x-ray intensity and the x-ray spectrum.^61^ That these considerations had no base was shown elegantly by a TRX experiment with PYP microcrystals^62^ (Fig. 1). Due to the serial fashion the crystals were provided and the femtosecond XFEL pulses that probed the progress of the reaction, the method has been called time-resolved serial femtosecond crystallography (TR-SFX).^62,63^ This experiment opened the door for the real-time observation of the *trans* to *cis* isomerization itself.

In 2016, TR-SFX results were published that report the first structural characterization of any *trans* to *cis* isomerization using PYP as a model system.^31^ The wildly impossible goal of 1997, namely, to explore how the pCA chromophore achieves the isomerization, has been reached almost 20 years later. Figure 2 shows snapshots from a time-course of nine structures from 140 fs to 3 ps that includes the isomerization reaction at around 600 fs. In the first few hundred fs PYP relaxes on the excited state potential energy surface (PES). The pCA chromophore is still in the *trans* configuration. After this, structures in twisted *cis* configurations are observed that relax toward the first stable intermediate I_T_. This has been largely interesting to theorists who predicted, already a decade earlier, that the *trans* to *cis* transition proceeds through a conical intersection^64^ (Fig. 2), which is a point or a seam where the electronic ground state PES and the excited state PES meet.^64^ The TR-SFX data were further analyzed by a combination of manifold embedding and quantum mechanical calculations.^32^ The transition through the conical intersection (Fig. 2) was pinpointed further to 615 fs and modeled by wave-packet dynamics on the electronic excited state PES near the conical intersection.^32^ This convincingly ties excited state protein structural dynamics to quantum mechanical considerations. More experiments with PYP are conceivable, for example, by using optical control and pump-dump-probe sequences to explore coherent dynamics^65^ on the ground state and excited state PESs.

## TR-SFX EXPERIMENTS ON PHYTOCHROMES

**FIG. 4.**
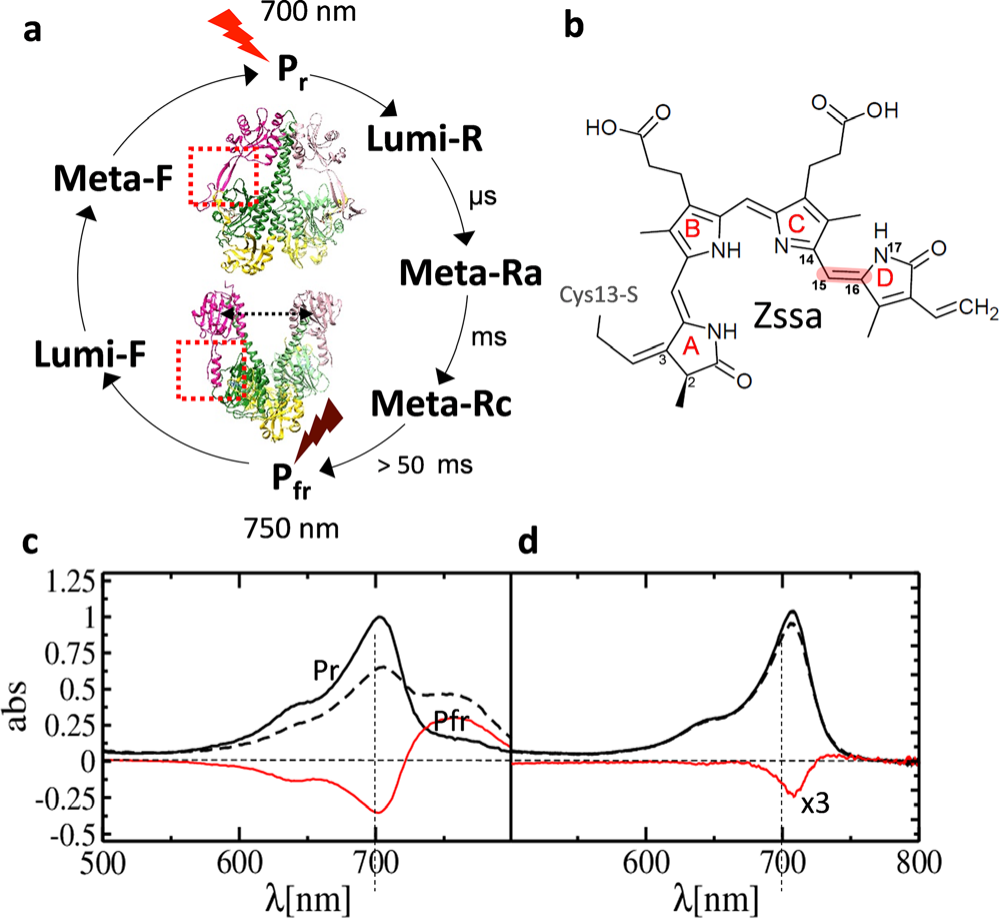
Phytochrome photocycle, and UV-vis absorption spectra of *Sa*BphP2. (a) The phytochrome photocycle and approximate time scales for intermediate (Lumi-R, Meta-Ra, and Meta-Rc) formation are shown. Pfr is formed after about 50 ms, with variations between the species. The two half cycles can be driven by illuminating the stable Pr and Pfr states (displayed for the *Sa*BphP2 and *Dr*BphP PCMs, respectively) by red and far-red light. The sensory tongue is marked by a dashed box in each structure. (b) The chemical structure of BV bound to Cys-13 in the phytochrome. The double bond Δ15,16 about which ring D rotates is marked. In Pr, the structure is all-*Z* syn-syn-anti. (c) *Static* absorption spectra of the *Sa*BphP2 Pr (solid line) to Pfr (dashed line) transition in solution. The difference is shown in red. (d) As (c) but in the crystal. The difference (red line) is enhanced threefold.

In the summer of 2005, the decision was made in the Moffat lab to apply TRX to new, more complex systems that involve light-triggered enzymatic reactions. Red-light photoreceptors, known as phytochromes, were one of these systems. The phytochromes are conserved from bacteria to plants. In plants, they regulate essential developmental stages such as seed germination and shade avoidance.^66^ Phytochromes are red/far-red light photoreceptors that usually contain a C-terminal enzymatic domain (ED), such as a histidine kinase that is regulated via a reversible photocycle with several intermediates^67^ [Fig. 4(a)]. Bacteriophytochromes (BphPs) were first discovered in 1999^68,69^ and regulate the synthesis of light-harvesting complexes in photosynthetic bacteria, while their role in non-photosynthetic bacteria is not so well-understood. Recently, links to fruiting body formation of myxobacteria,^70^ swarming motility,^71^ and carotenoid synthesis^72^ were reported. BphPs consist of PAS (Per-Arndt-Sim), GAF (cGMP phosphodiesterase/adenylyl cyclase/FhIA), and PHY (phytochrome-specific GAF-related) domains that make up the photosensory core module (PCM) and a covalently attached enzymatic domain (ED) with usually histidine kinase activity. It is believed that light modulates the activity of the ED which, in turn, leads to an important physiological response of the bacteria via a two-component signaling pathway. Here, the histidine kinase phosphorylates a downstream response regulator (RR) that ultimately triggers gene expression.^73^ BphPs have a biliverdin (BV) chromophore attached to a conserved cysteine residue in the PAS domain. The BV is photoactive and changes the configuration of its ring D by a 180° isomerization about the Δ_15,16_ double bond [Fig. 4(b)] upon absorption of red light. The reaction is reversible by exposure to far-red light [Fig. 4(a)]. The light-triggered isomerization results in a conformational change of the protein that, through about 150 Å, regulates the ED activity. Although PHYs were discovered nearly half a century ago, the structures of these proteins were completely unknown until 2005 when the first PHY crystal structure of a fragment of the *Deinococcus radiodurans* BphP containing the chromophore binding domain (CBD) was determined.^74^ At about the same time, truncated variants of a BphP constructs from the photosynthetic purple bacterium *Rhodopseudomonas palustris* were cloned and purified in the Moffat lab.

*R. palustris* contains five BphPs, first characterized by Giraud *et al.* (2005). The structure of RpBphP3 CBD was determined and published in 2007 to become the second structure of any PHY determined to that point.^75^ Interestingly, RpBphP3 is the only PHY to date to undergo red/near-red light absorption. Although it binds the same BV chromophore and has high sequence similarity to classical BphPs, including RpBphP2, it does not form a Pfr state upon red-light absorption. Instead, it shifts to a near-red light absorbing state, denoted as Pnr.

One year later, the crystal structure of a bathy BphP from *Pseudomonas aeruginosa* was determined.^76^ Bathy BphPs exist in the Pfr state in the resting state. When excited by far-red light, they transition to the Pr state. The structure of the entire PCM (with PAS-GAF-PHY domains) from the non-photosynthetic bacterium *P. aeruginosa* was published^76^ jointly with a cyanobacterial Cph1 PCM.^77^ The cyanobacterial Cph1 binds phycocyanobilin (PCB) instead of BV. Both PCM structures were determined in the respective dark-adapted states of each protein (Pfr for PaBphP and Pr for Cph1). It was noted that the PHY domains undergo significant conformational changes during the Pr to Pfr transition.

A preeminent feature of the PCM was a stretch of about 50 amino acid residues called the sensory tongue that extended from the PHY domain toward the chromophore binding pocket [dotted boxes in Fig. 4(a)]. Within the sensory tongue, a conserved PRXSF motif^78^ shows a significant switch from a β-strand in the Pr state of Cph1 to an α-helix conformation in the Pfr state of PaBphP. The PRSFX motif was highlighted in a major paper in 2015 by Moffat and members of his research lab, where PCM structures of RpBphP2 and RpBphP3 were published,^78^ both determined in the Pr state. It was noted that the Pnr-forming RpBphP3 lacked a critical proline in the PRXSF motif and contained a threonine instead. It was the only BphP with such a distinct feature. A single amino acid change, proline in place of threonine, resulted in the conversion of the RpBphP3 mutant to a classical BphP with Pr and Pfr absorbing states. These results demonstrated how important the sensory tongue is in regulating the photoactivity of the BV-binding BphPs although none of the amino acids from this region formed covalent interactions with the BV chromophore. In addition, the sensory tongue's β-strand to α-helix conversion was revealed by the first crystal structure of the light-illuminated Pfr state of the classical DrBphP PCM.^79^ Although determined at 3.8 Å resolution, this secondary protein structural change was clearly visible, together with a separation of the PHY domains [Fig. 4(a)]. Interestingly, in the Pfr-structure of the bathy PaBphP, the separation of the PHY domains was not observed.

The race for a room temperature TRX experiment began, requiring highly diffracting and photoactive BphP crystals. Unfortunately, the CBDs of truncated BphPs are only transiently photoactive since they lack the PHY domain and the corresponding sensory tongue. They revert to the reference state within a few nanoseconds (2 ns).^80^ The PCMs form stable photoproducts, but the crystal quality was low and the reaction irreversible. These practical challenges prevented investigations on the BphPs by the (synchrotron based) Laue method. In the meantime, a collaboration of Moffat with John Kennis, a time-resolved ultrafast spectroscopist, offered insight into the photocycle intermediates of RpBphP2^81^ and RpBphP3.^82^ New BphP were discovered in myxobacteria by the lab of Stojković (a former postdoctoral scholar of Moffat)^83^ who also cloned, purified, and crystallized the myxobacterial BphPs. Myxobacteria are non-photosynthetic soil bacteria unique amongst prokaryotes for their macroscopic multicellular fruiting bodies, formed upon starvation and in the presence of visible light. Although this photomorphogenic response has been known for nearly 50 years, the molecular mechanism remained unknown. Genome sequences became available at the turn of the millennium. Putative photoreceptor genes such as BphPs and PYPs were discovered in all three suborders of myxobacteria which provide clues about the signaling pathways in the myxobacteria.

**FIG. 5.**
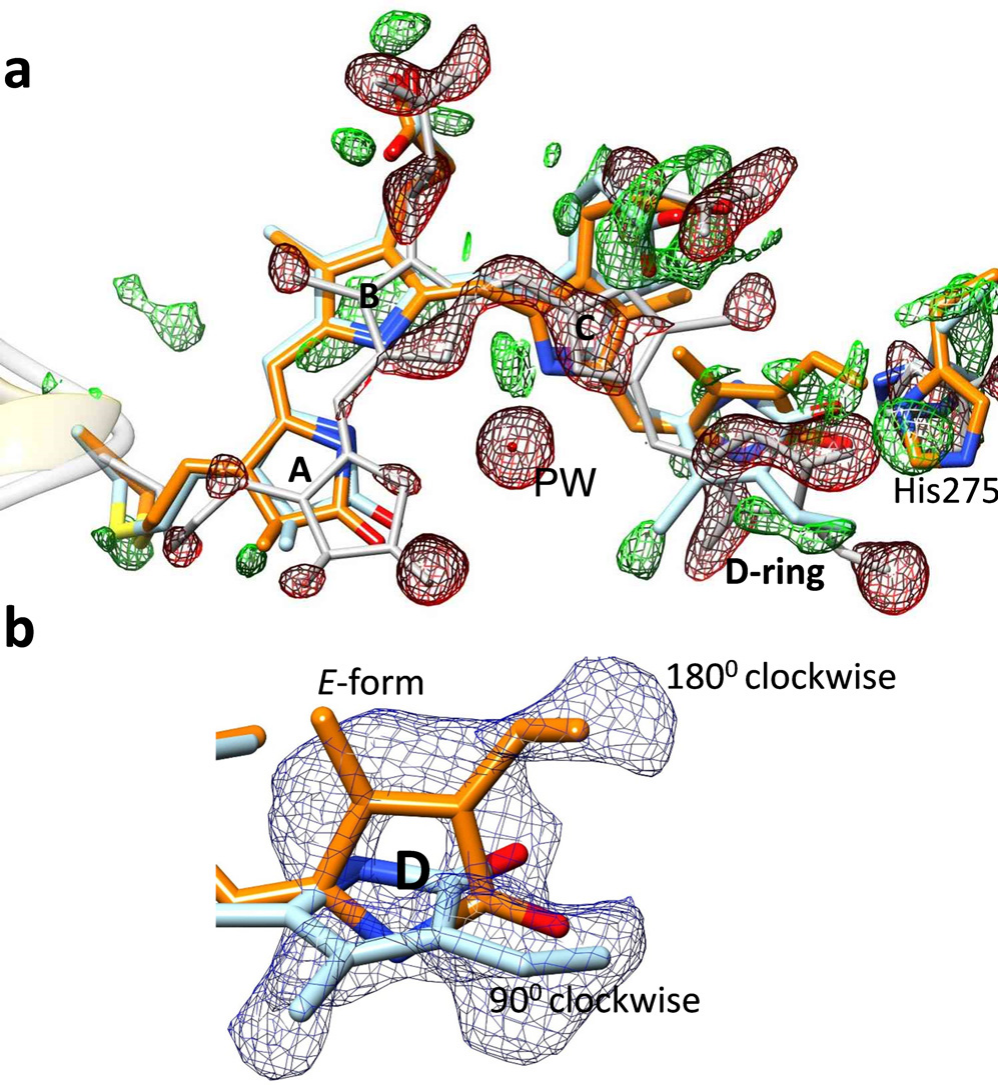
Chromophore displacements and D-ring rotations.^45^ (a) Overall biliverdin chromophore configuration in subunit A at the 33 ms time delay. Gray: reference (dark), orange: intermediate at 33 ms. subunit A, reference structure in gray, the result of the clockwise D-ring rotation is shown in light blue, the fully isomerized structure is shown in orange. The difference electron density is displayed in red and green, contour levels: red, −2.7 σ, green, 2.7 σ. (b) Conventional electron density (blue) showing the isomerization. Structures are colored as above.

With the development of SFX at XFELs, BphPs could be structurally investigated to high resolution.^70,84–86^ The first successful (ambient temperature) TR-SFX experiment was performed on the CBD of the *Deinococcus radiodurans* BphP.^87^ The BV ring-D rotated anticlockwise by about 50°. The important pyrrole water [Fig. 5(a)], which stabilizes the A, B, and C-rings of the BV chromophore in the dark-adapted and light-illuminated states of BphPs,^79,88^ has been photoejected. Small changes in the chromophore binding pocket already provided hints how the signal could migrate to the more distant parts of the protein and to the effector domains. The relatively small anticlockwise ring-D rotation was unexpected, as a complete rotation (by 180°) has been hypothesized.^89^ However, the lack of the sensory tongue from the PHY domain resulted in an open chromophore pocket, exposed to the solvent. This could potentially provide insight why the CBD constructs were only partially photoreactive.

A conclusive result was obtained from highly diffracting (1.5 Å resolution in the Pr-state) and photoactive PCM crystals of a classical myxobacterial BphP from *Stigmatella aurantiaca*. Two different BphPs (SaBphP1 and SaBphP2) were isolated from S. *aurantica*, and the structures of their PCMs and several mutant variants including shorter CBD constructs were solved.^70,86^ SaBphP2 microcrystals are photoactive in the crystals [compare Figs. 4(c) and 4(d)]. They were used for TR-SFX experiments at the Japanese XFEL SACLA in a collaboration of the two authors of this article (both former postdocs of Moffat). Their expertise brought together a larger international team, including undergraduate and graduate students from Northeastern Illinois University (NEIU) a primarily teaching and Hispanic Serving Institution (HSI) in Chicago IL. The crystals were illuminated by 4 ns pulses of 640 nm laser light and probed with XFEL radiation.^45^ These experiments resulted in the first successful time-resolved structure determination of a PCM of any BphP. Structures obtained at 5 ns and 33 ms after reaction initiation were examined. The rotation of the D-ring by 180° in a clockwise direction and the ejection of the pyrrole water was observed in these intermediates [Figs. 5(a) and 5(b)]. Although the sensory tongue (including the PRXSF motif) has moved so that the important salt bridge between the conserved arginine (R) and the conserved aspartate (D) in the PASDIP motif of the GAF domain has been broken, the conversion from the beta-sheet to an alpha helix was not captured, most likely due to limitations of the protein relaxations within the tight crystal lattice. Solution experiments perhaps with TR-cryoEM^90–92^ and/or single particle x-ray diffraction^35,93^ will further shine light on this transition.

In conclusion, Moffat initiated several research directions with proteins that are suitable for TRX experiments. Numerous publications in highest-ranking journals^16,22,94,95^ witness the great success made possible by decades of diligent technology developments mentioned above. Several of these projects were significantly extended by collaborators and elevated to new heights at XFELs^31,45,62,87^ and with cryo-electron microscopy (EM).^96^

## Data Availability

Data sharing is not applicable to this article as no new data were created or analyzed in this study.
